# Supplementary material for: Dynalign II: common secondary structure prediction for RNA homologs with domain insertions
Source: Nucleic Acids Res. 2014 Nov 21;42(22):13939–48. doi: 10.1093/nar/gku1172 (PMC4267632; doi:10.1093/nar/gku1172)
Supplement: SUPPLEMENTARY DATA [file supp_gku1172_nar-02021-z-2014-File012.zip › manual/Overview/Updates.html]

RNAstructure Installation and Overview: Repository Updates and History


|  |  |  |
| --- | --- | --- |
|  | RNAstructure Installation and Overview Package Updates and History | - Contents - Index |
| **RNAstructure 6.0**   1. Fixed a bug where specifying a constraint file (in any program or OS) was removing prior unpairing constraints specified in a sequence file. 2. Fixed a bug in ShapeKnots. While filling in dingle or double mismatches it created new non-canonical pairs, which led to giving large positive energies to those structures. 3. Improved ShapeKnots speed by removing boundaries checking in vectors. 4. Fixed a bug in the Windows GUI for bimolecular folding where internal loops were intermittently being forbidden. 5. Change the Macintosh compiler flags to match new Clang compiler. Also, revised code to match the fact that OpenMP is absent from Leopard and Clang.   **RNAstructure 5.6 (7/31/2013)**   1. Added Multifind to package. 2. Added parallel processing (SMP) versions of bifold, bipartition, efn2, Fold, MaxExpect, oligoscreen, partition, and stochastic. These programs take advantage of multicore computing environments. 3. The Windows GUI now runs in parallel on multicore computers for predicting lowest free energy structures, calculating partition functions, calculating free energy changes, oligoscreen calculations, and stochastic sampling. 4. Fixed bug where command line programs were not recognizing long flags without associated parameters. This also affected the web servers. 5. Fixed a bug with reading HMM alignment parameters for Dynalign calculations in the Windows GUI. This was causing the program to crash for Dynalign calculations. 6. Fixed a bug where bootstraped restraints were not being correctly calculated in Fold.   **RNAstrucure 5.5 Update (5/16/2013)**   1. Uploaded a new version of the Windows 64-bit graphical interface. This fixes problems with launching the program from the start menu.   **Online Documentation Update (4/16/2013)**   1. Added instructions for using Windows 8.   **RNAstructure 5.5 (4/5/2013)**   - New Programs  1. Added ShapeKnots as text interface. 2. Added GPU version of partition function (partition-cuda) as text interface.  - Algorithms   1. Fixed minor memory access issues in NAPSS program.   2. Fixed an error in Multilign where SHAPE intercept and SHAPE slope weren't being set properly. - JAVA GUI  1. Fixed bug in JAVA GUI where opening .seq files from disk would sometimes fail.  - Code Base   1. Updated Makefiles to build more efficiently and standardized their formats.   2. Simplified text interface parsing machinery and made all flags case-insensitive.   3. Cleaned up format, comments, and organization in all text interfaces that use the standard parsing machinery. - Data tables/thermodynamics/parameters   1. Updated dnacoaxstack.dat. Fixed problem where a subset of mismatch-mediated coaxial stacks were missed in DNA.   2. Fixed bug where not all special DNA tetraloops were being read from disk.   3. Updated default SHAPE slope and intercept for all programs.   4. Changed thresholds for SHAPE color annotations.   5. Added support for knowledge-based free energy terms for stacking restraints in Fold. - Drawing Framework   1. Reduced the size of dot plot labels so they didn't run together in certain instances.   2. Made a small change to dot plot layout so dots at the first nucleotide index always fall on the first grid line.   3. Fixed a bug where sometimes plot bounds weren't set right, so some dots weren't shown.   4. Fixed a bug where sometimes the drawing windows wouldn't resize properly when dragged.   5. Added the ability to draw structures with their backbone stretched out in a straight line.   6. Improved drawing of structures so large structures are drawn faster, and move and zoom more efficiently.   7. Fixed a glitch in CircleCompare where sometimes the probability annotation for the accepted structure was being shown for all structures, not for each predicted structure.   8. Fixed a glitch in CircleCompare where probability annotation was not always consistent in pairs.   9. Added distinct flags to CircleCompare so the user can annotation with either predicted or accepted structure probabilities.   10. Added the ability for structure drawing programs to read probability information from dot plot text files as -log10(Probability).   11. Added the ability for ProbabilityPlot to read dot data from a standard dot plot file. - Documentation   1. Fixed mistake in TurboFold documentation where the SHAPEslope and SHAPEintercept default values were reversed. This reversal was only in the documentation.  **Windows Text Interface Executables Update for RNAstructure 5.4 (09/05/2012)**   1. Posted a new version of .zip files (both 32- and 64-bit), which fixes an error with ProbabilityPlot, EnergyPlot, and DynalignDotPlot where these programs would not work on all Windows Computers.  **Web Servers (08/28/2012)**   1. Improved CT file reading by making it more robust to differences in format.  **Mac OS X Executables Update for RNAstructure 5.4 (08/23/2012)**   1. Fixed a bug where the Java GUI didn't always run properly. Current executables run on Snow Leopard, Lion, and Mountain Lion.  **Web Servers (08/13/2012)**   1. Fixed bug where pasted sequences without new lines at the end sometimes caused calculation errors.  **Web Servers (08/10/2012)**   1. Fixed bug in multiple sequence servers where a FASTA file wasn't being uploaded and split into multiple sequences properly. 2. Changed wall time of run scripts from one hour to one day.  **Web Servers (08/09/2012)**   1. Clarified error message that occurs when no sequence input at all is given to a web server that takes sequence input. 2. Fixed a bug in the Predict a Secondary Structure server where the calculated partition function save file wasn't being used for all calculations. 3. Fixed a bug in the Multilign and TurboFold servers where the proper error page wasn't being shown if too few or too many sequences were given.  **Web Servers (08/08/2012)**   1. Fixed broken links and style sheet references on page that shows up if structures have no pairs.  **RNAstructure 5.4 (08/02/2012)**   1. Added webservers for all programs. 2. TurboKnot added to GUI as an option in TurboFold. 3. Breakage of DNA pseudoknots, as well as pseudoknot breakage options, added to JAVA GUI. 4. Added 64-bit executables as downloads for Windows. 5. Revised Windows GUI to conform with Windows 7 standards. This also fixes issues with drawing dot plots from text files and issues with showing oligonucleotide self-structure from the OligoWalk interface. 6. Fixed a bug in the Windows GUI, where pressing the F1 key resulted in a "Failed to Launch Help" window in Windows XP and later. 7. Fixed command line bug in bifold interface where the intramolecular flag did not always forbid intramolecular pairs as it should have. 8. Fixed command line bug in text interfaces where whole numbers specified with zeros after a decimal point were sometimes rejected as non-numeric. Negative numbers were also sometimes rejected as non-numeric. 9. Corrected the documentation for the Dynalign text interface, where the default "DNA = false" should have been written as "DNA = 0." Also, to set DNA folding, the user should specify "DNA = 1." 10. Fixed a typo in miscloop.dat and dnamiscloop.dat tables in data\_tables directory. The file stated "miscloop asym," but now states "multiloop asym." This value is the multibranch loop asymmetry penalty that is used by efn2. This did not affect structure prediction or energy calculations, and the change was made to simply clarify what thermodynamic parameter this is. 11. Corrected bug in JAVA GUI where the partition function bimolecular module sometimes overwrote its second input sequence file, rather than using the desired output name. 12. Corrected bug in JAVA GUI where file chooser extension sanity checks sometimes failed. 13. Added an RNAstructure\_java\_utilities folder to facilitate expansions in JAVA capabilities. 14. Re-implemented internal workings of the JAVA GUI to aid in organization and comprehension. 15. Fixed a bug in Dynalign, where conserved 3' dangling ends on helices at exterior loops were not allowed. 16. Fixed a bug in extrapolation of free energies to temperatures other than 37 degrees C. 17. Fixed a dot plots bug where dots at the boundaries of color ranges sometimes showed up as the wrong color or disappeared. 18. Reorganized Makefiles to build more efficiently and include explicit dependencies. 19. Standardized Postscript and SVG structure drawing so their default is always clockwise orientation. 20. Added a redone JAVA drawing framework, which includes the ability to create self-contained JAVA drawing programs for structures and dot plots. 21. Simplified the controls in the JAVA drawing framework so only the structure currently drawn can be exported to Postscript. 22. Added items where necessary to the JAVA drawing framework so structures and dot plots can be exported as both Postscript and SVG. 23. Fixed a bug in JAVA drawing framework where the default for structures wasn't clockwise orientation. 24. Removed "New Window" and "Arrange Icons" options, which did nothing, from the menus of the Windows GUI. 25. Added ability to switch between structures and zoom in the drawing framework using the command key (on Mac) as well as the control key. 26. Fixed the description of MAP mode in the PARTS text interface manual.  **RNAstructure 5.3 (05/10/2011)**   1. Added TurboFold to the package. 2. Fixed a bug where drawing suboptimal structures did not always work in the JAVA GUI for Mac and Linux. 3. Fixed a bug where bimolecular partition functions were missing some states (single dangling ends and no dangling ends) at helix ends. 4. Added the ability to include SHAPE data when calculating folding free energy changes with efn2 program. 5. Added the ability to apply folding restraints for the formation of specified base pairs. 6. Corrected Windows and JAVA graphical interfaces for OligoWalk to show the correct temperature of folding free energy changes when a user adjusts the temperature of the calculation. (Prior to this, free energies' changes were always listed as being at 37 degrees C.) 7. Added the Overview help pages to the manual. 8. Corrected bug in JAVA GUI where the message bar at the bottom of the screen didn't always update when a temperature menu was clicked. 9. Corrected bug in JAVA GUI in OligoScreen where file names were being refreshed incorrectly. Also switched oligomer type default to DNA, rather than RNA. 10. Corrected bug in JAVA GUI of OligoWalk where oligonucleotides were always RNA, even when DNA was selected.  **RNAstructure 5.2 (11/17/2010)**   1. Added Multilign to the package. 2. Fixed an issue when breaking pseudoknots, but leaving the maximum non-pseudoknotted pairs, where non-canonical pairs in the input structure were mistakenly broken. This was also an issue with the count of pseudoknotted pairs in the CircleCompare program. 3. Added support for writing ct files for long sequences (>9,999 nucleotides). 4. Removed GAIL rule from dnamiscloop.dat and dnamiscloop.dh. 5. Made the internal loop asymmetry penalty a single constant for DNA folding. This fixed an occassional bug with folding by DNA rules. 6. In Windows GUI only, changed default value for the minimum helix length in ProbKnot from 2 to 3, which is the preferred value. 7. Added color-annotation for single-stranded nucleotides in drawing programs. 8. Added the ability to use DNA parameters to the Dynalign command line interface. 9. Added the ability to change the folding temperature in the Dynalign command line interface. 10. In the Windows GUI, set the default file choice to .ALI when clicking the "Alignment File" button. 11. In Dynalign, revised the model for using alignment data to limit the extent of calculation. This can lead to a different set of suboptimal structures than previous versions. This also fixes a traceback error encountered in rare circumstances. 12. Added the EnsembleEnergy program to the command line interface programs. 13. Improved the font in the html help for the GUI.  **RNAstructure 5.1 (06/30/2010)**   1. Changed the thermodynamic parameter for adding an additional helix to a multibranch loop ("helix penalty" in miscloop.dat). The value is now set to -0.6 kcal/mol, which is the value determined by a linear regression fit to experimental data. This value provides slighly higher average accuracy for maximum expected accuracy structure prediction (MaxExpect and ProbKnot programs). This change means that predicted structures might be different than those predicted by previous version of RNAstructure. Base pair probabilities, however, should be highly similar to predictions from previous versions of the program. 2. Added ProbKnot program to text interfaces and GUIs. 3. Added online help for text (command line interfaces). 4. Restructured downloads so that the text interface binaries now also come with complete repository, including help and examples. 5. Added circular drawings to GUIs for drawing structures that contain pseudoknots. 6. Added the ability to read FASTA-formatted sequence files. 7. Updated online help for GUIs to better reflect the current state of the software. 8. Fixed problems with Dynalign dot plots. 9. Fixed problem reading SHAPE data in JAVA GUI.  **RNAstructure 5.03 (03/22/2010)**   1. Added the automatic generation of a title for new sequences in the GUIs (both Windows and JAVA). This fixes a problem where RNAstructure fails to recognize a sequence because it contained no title. 2. Added CircleCompare (comparision of two secondary structures, including pseudoknots). 3. Fixed the grayed "forbid unimolecular pairs" menu option when performing bimolecular folding with the Windows GUI.  **RNAstructure 5.02 (01/22/2010)** 1. Revised PARTS to make it more user friendly. 2. Fixed problem where precompiled text interfaces could not find the shared library on some machines and therefore not run.  **RNAstructure 5.01 (12/30/2009)** 1. Added the ability to read FASTA-formatted sequences to text interfaces. 2. Added the Dynalign Intel C++ project for Microsoft Visual Studio to download. 3. Added the Fold Intel C++ project for Microsoft Visual Studio to download. 4. Changed the default parameters for SHAPE data pseudo energies to match best practices. 5. Cosmetic changes made to a number of the text interfaces. 6. Fixed a problem where text interfaces on Linux and Mac could not find the shared library and therefore did not run.  **RNAstructure 5.0**   1. 09/30/2009: New major update of RNAstructure. This version has a graphical user interface in JAVA for use on Mac and Linux machines. Text interfaces are now available for DOS, Linux, and Mac OS X. A class library is available for programmers. All source code is available for download. 2. 10/03/2009: New Windows text interface binaries posted. This fixes the problem that manifests with the error "This application has failed to start because the application configuration is incorrect," which appeared on some computers. | | |
| Visit The Mathews Lab RNAstructure Page for updates and latest information. | | |
